# The Burden of Illness of Treatment-Induced Vasomotor Symptoms in Individuals with Breast Cancer: A Systematic Literature Review

**DOI:** 10.3390/jcm14082601

**Published:** 2025-04-10

**Authors:** Antonia Morga, Aki Shiozawa, Lora Todorova, Mayank Ajmera, Maria Arregui, Erika Wissinger

**Affiliations:** 1 Astellas Pharma Europe Ltd., Addlestone KT15 2NX, UK; 2 Astellas Pharma US, Inc., Northbrook, IL 60062, USA; 3 Cencora, 30159 Hannover, Germany; 4 Cencora, Conshohocken, PA 19428, USA; erika.wissinger@cencora.com

**Keywords:** vasomotor symptoms, hot flashes, night sweats, breast cancer

## Abstract

**Objective:** This systematic literature review (SLR) evaluates the global burden of treatment-induced vasomotor symptoms (VMSs) in individuals with breast cancer receiving tamoxifen or aromatase inhibitors (AIs). **Methods:** Embase and PubMed were searched for observational and interventional studies published between January 2010 and January 2023 reporting on adults who experienced moderate to severe VMSs after tamoxifen or AI treatment for breast cancer. Epidemiological, clinical, humanistic, economic, and treatment pattern data were extracted where available. **Results:** Of 694 unique publications identified, 37 independent studies (22 observational and 15 interventional) were included. The prevalence or incidence of treatment-induced VMSs was reported in 17 studies. The prevalence of hot flashes ranged from 32.5% to 82.9% in observational studies, while their incidence ranged from 2% to 60.0% in interventional studies. In four studies that reported data, individuals experienced VMSs with a frequency of 2 to 20 episodes per day. There were limited data on VMS timing (within a 24 h period or in relation to treatment dosing), duration, and correlations with clinical outcomes. Age, weight gain, body mass index, ethnicity, employment intensity, and certain genetic haplotypes were identified as risk factors for VMSs; however, these factors were often reported in only one study each. Notable evidence gaps in the literature included treatment options or management strategies for treatment-induced VMSs and the economic burden associated with treatment-induced VMSs. **Conclusions:** This SLR highlights the burden of treatment-induced VMSs in individuals with breast cancer receiving tamoxifen or AI therapy. Moderate to severe symptoms were reported in a large proportion of individuals across several studies. Evidence gaps were identified for economic burden and treatment patterns; further research is needed to understand the unmet needs for this population.

## 1. Introduction

Breast cancer is the most commonly diagnosed cancer among women, with an estimated 2.3 million new cases worldwide in 2022 [1]. Mortality rates for breast cancer have fallen in recent years due to early diagnosis and better treatments [2]. As survival rates improve, there is a greater focus on managing the long-term adverse effects of therapy for breast cancer survivors.

Adjuvant endocrine therapies such as tamoxifen, a selective estrogen receptor modulator, and aromatase inhibitors (AIs) such as anastrozole, letrozole, or exemestane are often administered as maintenance therapy to individuals with breast cancer who have completed curative treatment [3]. Despite the benefits of these therapies in promoting disease-free survival among breast cancer survivors [4], estrogen deprivation as a result of maintenance therapy with tamoxifen or AIs may lead to treatment-induced vasomotor symptoms (VMSs), which consist of hot flashes and night sweats [5]. Hot flashes range from a transient warming sensation to a sudden feeling of intense heat, profuse sweating, and flushing, while night sweats are excessive sweating during sleep. VMSs can have a substantial effect on health-related quality of life (HRQOL), negatively impacting sleep, mood, and cognitive functioning and leading to social impairment, work-related difficulties, anxiety, and fatigue [6,7].

In the general population, VMSs can be managed with hormone therapy. However, the use of hormone therapy in breast cancer survivors may lead to an increased risk of disease recurrence, particularly in those with estrogen-responsive breast cancer; as such, many individuals have concerns about using hormone therapy and are unwilling to take the treatment [8]. There are no standardized treatment guidelines for VMSs in individuals with breast cancer and there remains a lack of consensus on evidence-based interventions to treat VMSs in this population [9].

Despite the impact of VMSs on HRQOL [7], the global burden of VMSs in breast cancer survivors is not as well understood. This systematic literature review (SLR) was designed to assess the incidence, risk factors, and humanistic and economic burden of VMSs in individuals with breast cancer who were receiving maintenance therapy with tamoxifen or AIs.

## 2. Methods

### 2.1. Literature Search

This SLR was conducted according to a predefined protocol and in accordance with the Preferred Reporting Items for Systematic Reviews and Meta-Analyses (PRISMA) 2020 statement [10]. The Excerpta Medica database (Embase) and MEDLINE were searched via OVID to identify observational and interventional studies published between January 2010 and January 2023 reporting on adults who were experiencing moderate to severe VMSs after receiving maintenance hormonal therapy for breast cancer. The search strategy used EMTREE (Elsevier’s life science thesaurus) terms as well as free-text words specific to the population of interest (Table S1). The reference lists of relevant SLRs and meta-analyses we identified were hand-searched for additional citations of interest not captured by the database searches. In addition, abstracts from meetings of the following relevant conferences were searched from January 2020 to January 2023: European Society for Medical Oncology (ESMO) Congress, ESMO Breast Cancer, American Society of Clinical Oncology Annual Meeting, International Society for Pharmacoeconomics and Outcomes Research (ISPOR) Annual International Meeting, ISPOR Annual European Congress, and World Congress on Breast Cancer. The following health technology assessment (HTA) agency reports were also reviewed: National Institute for Health and Care Excellence, Canadian Agency for Drugs and Technologies in Health, Federal Joint Committee/Gemeinsamer Bundesausschuss, Haute Autorité de Santé, Institute for Clinical and Economic Review, and Agency for Healthcare Research and Quality. Review of the HTA reports was limited to those published up to 2020. The SLR was not registered.

### 2.2. Study Selection

Studies were screened for inclusion based on the prespecified population, intervention, comparator, outcome, and study design (PICOS) framework using the following criteria:

- Population: adults (≥18 years of age) with breast cancer who were receiving maintenance hormonal therapy (e.g., tamoxifen or AIs) for breast cancer and were consequently suffering from moderate to severe VMSs;
- Intervention: any treatment or no treatment;
- Comparator: any comparator or no comparator (i.e., single-arm studies);
- Outcomes: epidemiological, clinical, humanistic, economic, and treatment patterns (specific outcomes are presented in Table 1).
- Study design: clinical studies, registries, cross-sectional surveys, retrospective database studies, prospective observational studies;
- Language: English, German, or French.

A preliminary list of included studies was generated following title and abstract review with subsequent retrieval of full-text articles. Full-text review was then conducted to narrow the results to the final list of included studies. The reference lists of any included systematic reviews and meta-analyses were also checked against the final list of included studies to determine if any additional studies should be included in the SLR. One reviewer screened title/abstract and full text, and a second independent reviewer performed quality checks, including the screening of 20% of excluded studies.

### 2.3. Data Extraction

For studies that met eligibility criteria, patient demographic and outcomes data were extracted (as available) into a data-extraction template developed in MS Excel^®^. Data were extracted by 1 reviewer and quality checks, including validation of 50% of the extracted data, were conducted by a second independent reviewer. Multiple publications that were identified for the same study, population, and setting that reported data for the same intervention were linked and reported as a single study.

## 3. Results

### 3.1. Search Results

**Figure 1:**
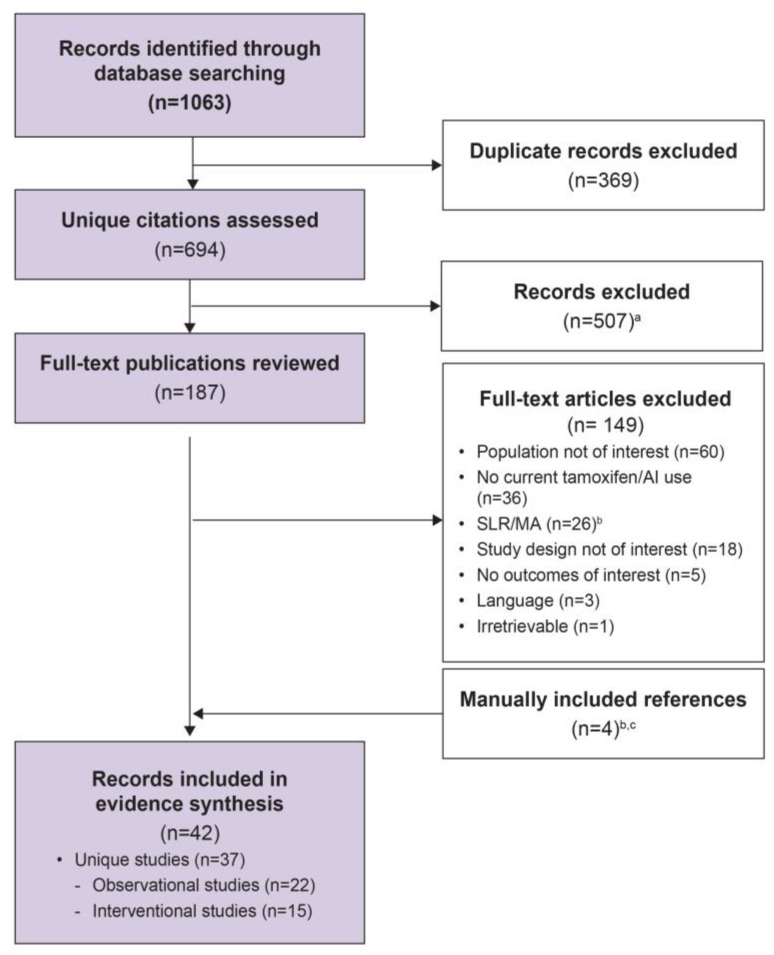
PRISMA flow diagram. AI, aromatase inhibitor; MA, meta-analysis; SLR, systematic literature review. ^a^ Records did not meet inclusion criteria. ^b^ The reference lists of SLRs were manually searched for any additional studies; three studies were identified for inclusion. ^c^ One additional study was manually identified for inclusion from a health technology assessment website.

The literature search identified 1063 citations, of which 694 unique citations were screened at the title and abstract level. Of these, 187 citations underwent full-text review, and 38 were included as shown in the PRISMA flow diagram (Figure 1). An additional three records for inclusion were identified by bibliography checks, and one was manually identified on an HTA website. In total, 42 publications reporting on 37 independent studies were included in the SLR (Table S2). Across the 37 studies, 22 were observational and the remaining 15 were interventional. The number of studies that reported each outcome of interest, stratified by study design, is shown in Table 2.

### 3.2. Study Characteristics

The 22 observational studies, conducted between 2002 and 2017, were heterogeneous in terms of study design, geographic location, sample size, and study population. These included prospective cohort studies (n = 10) [11,12,13,14,15,16,17,18,19,20,21], cross-sectional studies (n = 7) [22,23,24,25,26,27,28], retrospective cohort studies (n = 3) [29,30,31], a position statement (n = 1) [32], and a before/after–treatment observational study (n = 1) [33]. The observational studies were conducted in North America (n = 8) [15,16,18,26,28,29,30,31], the Asia–Pacific region (n = 7) [11,12,14,19,20,21,24,27], Europe (n = 4) [13,23,32,33], and Australia (n = 3) [17,22,25]. The sample size across the studies ranged from 10 [14] to 3595 [18] individuals.

Not all studies reported detailed characteristics of the study population. However, among the observational studies that reported data, the majority included postmenopausal individuals (n = 11 of 16 studies, 69%) with early-stage breast cancer (stage I–III) (n = 12 of 16 studies, 75%). The mean/median age was reported in 17 studies and ranged from 47 [14] to 68 [18] years. Only six studies reported data on race or ethnicity, and in these, White individuals comprised the majority (76% [30] to 100% [16]) of subjects. There were seven studies conducted in Asia that did not specifically report race or ethnicity; individuals from other racial and ethnic groups were underrepresented. Tamoxifen and anastrozole were the most common maintenance treatments for breast cancer in the observational studies.

The 15 interventional studies, primarily randomized controlled trials (RCTs), were conducted between 2001 and 2020 in Europe (n = 7) [34,35,36,37,38,39,40,41] or in North America (n = 4) [42,43,44,45,46] or were otherwise multinational (n = 4) [47,48,49,50]. The sample size ranged from 30 [42] to 9325 [49] individuals. Of the 15 interventional studies, the majority included postmenopausal individuals (n = 12 of 15 studies, 80%) with early-stage breast cancer (stage I–III) (11 of 15 studies, 73%). The mean/median age was reported in 12 studies and ranged from 53 [42] to 72 [39] years, and the majority (64% [44] to 95% [45]) were White individuals in the 6 studies that reported data; individuals from other racial and ethnic groups were underrepresented in the interventional studies. Tamoxifen, anastrozole, and exemestane were the most common maintenance treatments for breast cancer in the interventional studies.

### 3.3. Incidence and Prevalence of VMSs

**Figure 2:**
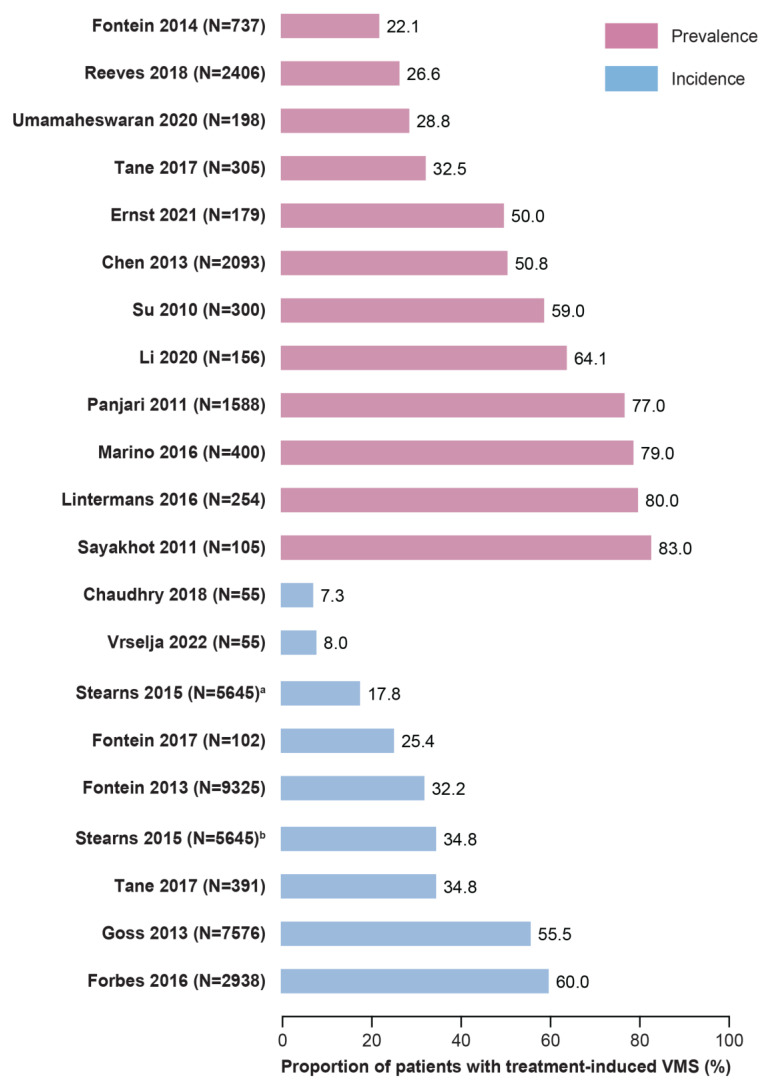
Prevalence and incidence of treatment-induced VMSs in individuals with breast cancer treated with tamoxifen or AIs. AIs, aromatase inhibitors; VMS, vasomotor symptom. Note: Numbers represent the highest percentage of incidence/prevalence reported for each study, regardless of whether they pertain to VMSs, hot flashes, or night sweats. For clinical trials, the total patient population is reported for the sample size, but the percentage is provided for the study arm with the higher percentage of events. ^a^ After 6 months. ^b^ After 12 months [11,13,15,17,18,19,20,22,25,26,29,30,38,39,45,46,47,49,50].

A total of 12 observational studies reported the prevalence and/or incidence of VMSs in individuals with breast cancer treated with tamoxifen or an AI (Table S3) [11,13,15,17,18,19,20,21,22,25,26,29,30]. The majority of studies with prevalence data (n = 7 of 11 studies, 64%) reported a prevalence of VMSs between 20% and 60% (Figure 2) [11,18,19,20,21,26,30]. The prevalence of hot flashes ranged widely from 32.5% after 12 months in a prospective study of 305 postmenopausal individuals treated with anastrozole [19] to 82.9% in a cross-sectional study of 105 individuals treated with adjuvant tamoxifen or AIs [25]. Similarly, the prevalence of night sweats varied considerably from 22.6% at 12 months in a prospective study of 305 individuals treated with adjuvant anastrozole [19] to 77% in a study of 105 individuals treated with adjuvant tamoxifen or AIs [25]. Among the four studies that reported data for both symptoms, the prevalence of hot flashes was consistently higher than that of night sweats [11,15,19,25].

The prevalence and/or incidence of VMSs was reported in six interventional studies [38,39,45,46,47,49,50]. The incidence of VMSs ranged from 17.8% after 6 months of treatment to 34.8% after 12 months, both reported in the same study of 5645 individuals treated with exemestane or anastrozole [46].

The incidence of hot flashes ranged between 2% and 60% in three interventional studies that reported data [45,47,50].

### 3.4. Risk Factors for VMSs

Risk factors associated with VMSs were reported in six observational studies [16,19,20,21,26,28,31] and included weight gain (>10 pounds) since breast cancer diagnosis [26] or increased body mass index [19] (Table 3). One cross-sectional study conducted in 300 postmenopausal individuals after an average AI exposure of 23 months reported that younger age was associated with an increased risk of occurrence of VMSs in univariate analyses and was a risk factor for hot flash severity in multivariate analyses (*p* < 0.001 for both) [26]. A probability model utilizing cross-sectional survey data from 360 individuals with early breast cancer treated with endocrine therapy who were experiencing VMSs found that persons who had 17 or more hot flashes per week or were aged between 49 and 63 years were more likely than not to report having severe symptoms on the Hot Flush Night Sweats Problem Rating Scale [28]. Regarding race, a US retrospective cohort study of electronic health records and patient-reported data reported more severe VMSs in Black individuals compared with White individuals [31]. Other factors that may contribute to VMS frequency and severity included physical or manually intensive employment [16] and variations in the *CYP19A1* gene (haplotypes) [20], which encodes for the aromatase enzyme and may affect the effectiveness or toxicity of AIs.

Four interventional studies reported data on risk factors for VMSs (Table 3) [38,40,43,45]. Younger age was associated with more severe VMSs [43], White race was associated with increased frequency of hot flashes (compared with non-White) [45], and *CYP19A1* gene polymorphisms were associated with an increased risk of VMSs [38].

### 3.5. Frequency and Severity of VMSs

A total of nine observational studies reported in 10 publications [14,15,19,23,24,26,28,31,33,34] and six interventional studies [37,38,42,43,44,50] reported on the frequency and severity of VMSs (Table S4). Across the five studies that reported data on frequency [34,37,42,44,50], the frequency of VMSs ranged widely, from an average of two episodes of hot flashes per day, in a cross-sectional survey of 295 individuals with early breast cancer undergoing endocrine therapy [28], to 20 VMS episodes per day, as measured by ambulatory skin conductance monitors in a study of 30 female breast cancer survivors treated with AIs or tamoxifen [42]. Additionally, this study highlighted a significant discrepancy between subjective (as reported in participant diaries) and physiologic VMSs (measured by ambulatory skin conductance monitors); it found that, on average, individuals reported only 46% of the total physiologic VMSs detected, illustrating a tendency to underreport VMSs [42].

Among individuals who reported VMSs, severity was generally moderate to severe as measured across several scales, including the visual analog scale, the Breast Cancer Prevention Trial Symptom Checklist, and patient reports. Studies that reported data on the frequency and severity of VMSs over time on maintenance therapy were conflicting, and no clear pattern was noted.

### 3.6. Clinical Outcomes

Few of the included studies (n = 3, 8.1%) reported correlations of VMSs with clinical outcomes and treatment discontinuation, and no data on the timing or duration of VMS episodes were identified across the 37 studies meeting eligibility criteria for this SLR. In two observational studies, experiencing VMS episodes was associated with a lower risk of recurrence among premenopausal individuals, as well as improved 5-year disease-free survival [11,12]. An interventional study conducted in 1485 postmenopausal individuals treated with exemestane observed longer relapse-free survival from 6 months after treatment initiation in individuals who experienced hot flashes than in those that did not [41]. In contrast, a retrospective analysis of data from a multinational interventional study of 7576 postmenopausal individuals with estrogen receptor (ER)- and/or progesterone receptor (PR)+ breast cancer treated with AIs found no association between VMSs and relapse-free survival [46].

### 3.7. Humanistic and Economic Burden of VMSs

Three observational studies [22,27,33] and five interventional studies [34,35,43,44,50] evaluated the humanistic burden of VMSs. A cross-sectional study of 804 individuals with breast cancer who were receiving anti-estrogen therapy found no difference in the severity of VMSs or endocrine-specific HRQOL between those individuals who had versus had not received prior adjuvant chemotherapy [22]. Another cross-sectional study of 280 individuals with early-stage breast cancer found that those individuals receiving adjuvant tamoxifen, compared with those not receiving tamoxifen, had significantly worse symptoms in the vasomotor domain of the Menopause-Specific Quality of Life Questionnaire (*p* = 0.0479) and experienced more severe VMSs [27]. An RCT in postmenopausal individuals with ER+/unknown primary breast cancer found no difference in endocrine symptoms over 5 years of treatment in individuals who switched from tamoxifen to exemestane versus continuing on tamoxifen [35]. Other data from the interventional studies demonstrated varying impacts of experimental treatments on VMS-associated HRQOL in individuals with breast cancer with no clear trends noted. However, gaps in the available evidence were identified for a number of areas, including economic burden, treatment patterns, VMS-associated breast cancer maintenance-therapy discontinuation, and correlations of VMSs with other clinical measures, such as HRQOL and breast cancer relapse or recurrence.

### 3.8. Treatment Patterns for VMSs

Overall, there was limited information in the literature about treatment for VMSs. Only three observational studies provided information on the current real-world management of VMSs in individuals with breast cancer [23,30,32]. There is a general lack of awareness and interventions to reduce the burden of treatment-induced VMSs. In a survey of 665 persons with breast cancer conducted in the UK, 40% of individuals reported that no healthcare providers had asked about their experience of VMSs, and only 26% of individuals had been offered treatment for VMSs [23]. In a US study of 89 individuals with ER+ and/or PR+ breast cancer receiving AIs, only 34% of individuals with VMSs received some form of symptom-palliating treatment, reflecting a lack of appropriate care for this condition [30]. Other studies identified in the SLR reported that acupuncture had a potential benefit on the frequency and severity of VMSs in individuals with breast cancer [14,33,34,44].

## 4. Discussion

This SLR identified published data on the burden of treatment-induced VMSs among individuals with breast cancer who were receiving tamoxifen or AIs as maintenance therapy. While some topics, such as the prevalence, frequency, and severity of VMSs in this population, were investigated in multiple studies, clear gaps remain in the available evidence, particularly around treatment patterns and the economic burden of VMSs for this population.

Prevalence data were well reported in the observational studies, and the majority (n = 10 of 15 studies, 67%) found that between 20% and 60% of individuals with breast cancer who were treated with tamoxifen or AIs experienced VMSs [18,19,20,21,26,30,39,45,47,49]. This wide range in the prevalence of VMSs may reflect differences in study populations, received treatments, VMS assessment methods, and the study design of the studies included in this SLR. Several potential risk factors for VMSs were identified in this SLR, including younger age [26], increased weight/body mass index [19], genetics [20], and manually intensive employment [16], but data for these risk factors were limited and often only reported in a single study. Data on the role of race and ethnicity as risk factors for VMSs were limited to two studies, and the results were conflicting [31,45].

Across the nine studies in this SLR reporting VMS frequency, rates were highly variable, ranging from two episodes per day [28] to 20 episodes per day [42]. This wide range in the frequency of VMSs may be reflective of the range of studies, with both observational and interventional studies included in this SLR. Furthermore, methods for detecting VMSs in the studies varied considerably, from self-reported VMSs to physiologic measures of VMSs such as skin conductance. One study found that individuals reported less than one-half of total physiologic VMSs [42], which suggests that patient-reported outcomes may greatly underestimate the frequency of VMSs. Although the frequency of VMSs was reported in several studies, data were lacking for the specific timing (i.e., within a 24 h period or in relation to treatment dosing) and for the episode duration of VMS events. Data were also limited for correlations between VMSs and other humanistic and clinical outcomes, such as HRQOL, disease recurrence, and treatment discontinuation. Conflicting data on the relationship between the occurrence of VMSs and breast cancer relapse or recurrence were reported in four identified studies. This may have been related to differences in the received treatments and the characteristics of the individuals across the studies [11,12,41,46].

No data on the economic burden of VMSs in breast cancer were identified in this SLR. This is notable, because VMSs associated with menopause, especially severe symptoms, have considerable economic burden due to direct and indirect costs associated with absenteeism, presenteeism, and decreased work productivity [51,52].

A better understanding of real-world treatment patterns for VMSs in breast cancer survivors is needed, as hormone therapy is generally contraindicated in this population. However, few studies reported data on the real-world management of VMSs for individuals with breast cancer undergoing maintenance therapy with tamoxifen or AIs. In the three studies that reported data, only 26% to 34% of individuals received some form of symptom-palliating treatment [23,30]. Furthermore, 40% of individuals reported they had not discussed their experience of VMSs with their healthcare provider, indicating they were not adequately prepared or offered treatment to ameliorate their symptoms [23]. Although acupuncture was observed to reduce the frequency and/or severity of VMSs in individuals with breast cancer and to improve patient-reported HRQOL [14,33,34,44], acupuncture may not be effective or accessible for all individuals.

The results of this SLR highlight the significant unmet need for managing VMSs among individuals with breast cancer receiving tamoxifen or AIs. There is currently no approved treatment or consensus for managing the symptoms of VMSs in this population. There is a need for more research on the management, impact on HRQOL, and economic burden of VMSs in this population.

### Limitations

Heterogeneity in the study population, study design, outcome measures and assessment, and treatment parameters made comparisons across studies difficult. In addition, information on the study period for many studies included in this SLR was missing (n = 7 (32%) observational studies and n = 6 (40%) interventional studies). This SLR identified only studies indexed in Embase or Medline or by hand search that were published between January 2010 and January 2023 in English, French, or German, and there is the possibility that other relevant studies were excluded or overlooked. The inclusion of additional databases or time periods may have increased the robustness of the search results. Text screening and data extraction were conducted by one reviewer, which may have introduced risk of bias in the selection and extraction of data. To minimize potential bias and errors, a second reviewer screened 20% of excluded studies and validated 50% of the extracted data.

## 5. Conclusions

This SLR highlighted the burden associated with treatment-induced VMSs in individuals with breast cancer receiving tamoxifen or AI therapy. VMS prevalence and frequency were commonly investigated, and several studies reported a large proportion of individuals had moderate to severe symptoms. The limited data on treatment patterns and lack of consensus on the management of VMSs illustrate a substantial unmet need in this population.

## Figures and Tables

**Table 1:** Screening criteria.

Inclusion Criteria		
Population	Adults (≥18 years of age) with breast cancer who are receiving maintenance hormonal therapy (e.g., tamoxifen or aromatase inhibitors) ^a^ for breast cancer and are consequently suffering from moderate to severe VMSs	
Intervention	Any/all/none	
Comparator	Any/all/none	
Outcomes	**Epidemiological:** Prevalence of VMSs Incidence of VMSs Demographics and risk factors, predictors of VMS frequency, and duration **Clinical:** Frequency, duration, and severity of VMSs (also at BL) ^b^ Hormone levels Symptom burden (generic and population-specific scales) Treatment discontinuation Comorbidities Correlations of VMSs with other measures	**Humanistic:** HRQOL (surveys, diaries, other nonstandard measures) Key results of other PROs (e.g., sleep-related outcomes, psychosocial outcomes) **Economic:** Direct costs Indirect costs HCRU Economic models (CEA, CUA, etc.) ICER/QALYs **Treatment patterns:** Prescribing patterns Treatment patterns, trends in treatment patterns
Study designs	Clinical trials, registries, cross-sectional surveys, retrospective database studies, prospective observational studies, modeling studies	
Language	Publications in English, German, and French were considered for inclusion	

BL, baseline; CEA, cost-effectiveness analysis; CUA, cost utility analysis; HCRU, healthcare resource utilization; HRQOL, health-related quality of life; ICER, incremental cost-effectiveness ratio; PROs, patient-reported outcomes; QALY, quality-adjusted life-year; VMS, vasomotor symptom. ^a^ Studies reporting results of interest for patient populations in which at least 75% of patients were receiving maintenance hormonal therapy were also included. ^b^ Including details on how these parameters were measured across studies.

**Table 2:** Number of observational and interventional studies by region and outcomes of interest.

Region/Outcomes	Observational Studies Reporting the Outcome of Interest (n = 22)	Interventional Studies Reporting the Outcome of Interest (n = 15)
Region		
North America	8	4
Asia–Pacific	7	-
Europe	4	6
Australia	3	-
Multinational	-	4
Epidemiological		
Prevalence/incidence of VMSs	12	6
VMS risk factors and associations	6	4
Clinical		
Frequency, duration, and severity of VMSs	9	6
Presence of pre-existing liver function abnormalities	0	1
VMS-associated breast cancer treatment discontinuation	3	3
Correlations of VMS with other clinical measures	3	3
Humanistic burden ^a^	3	5
Economic burden	0	0
Treatment patterns	1	2

VMS, vasomotor symptom. ^a^ Assessed using results from patient-reported outcomes surveys, diaries, or other nonstandard measures.

**Table 3:** Risk factors associated with VMSs.

Study	Country	Study Design	Population	N	BC Maintenance Treatment	Reported Demographics/Risk Factors and/or VMS Predictors
Observational (n = 6)						
Cole 2022 [28]	Canada	Gradient-boosted decision model to identify patients at risk of severe VMSs. The model incorporated cross-sectional survey data and considered 17 variables.	Early stage, pre/postmenopausal	360	Endocrine therapy (88.6%)	A threshold of 17 hot flashes per week was identified as being predictive of severe VMSs. Individuals aged 49 to 63 were more likely to report severe symptoms.
Hu 2022 [31]	US	Retrospective cohort	Early stage, menopausal status NR HR + BC	559 (30.1% Black; 69.9% White)	AET drug types during 1st yr: AI (anastrozole, exemestane, and letrozole): 57.7% Tamoxifen: 21.4% AI and tamoxifen: 20.8%	At BL, Black individuals reported more moderate-severity VMSs (i.e., ≥3 points on a 0–10 scale) than White individuals (1.2 vs. 0.7 items, *p* < 0.05). After 12 mos, Black individuals reported a greater number of VMSs with a ≥3-point increase during therapy ^a^ than White individuals (1.4 vs. 1.0 items, *p* < 0.05).
Nugent 2016 [16]	US	Prospective cohort	Stage I-IIIa, postmenopausal	49	Anastrozole: 100%	Individuals with physical or manual occupational roles had significantly more severe VMSs vs. those in technical, complex problem-solving roles (*p* = 0.028)
Su 2010 [26]	US	Cross-sectional	Postmenopausal individuals with a history of HR + BC	300	Anastrozole: 58% Letrozole: 23% Exemestane: 19%	Univariate analysis: Younger age (*p* < 0.001), shorter time since menopause (*p* < 0.001), smoking (*p* = 0.006), prior CT (*p* = 0.02), and prior tamoxifen therapy (*p* = 0.03) associated with higher risk of hot flashes. Multivariate analysis ^b^: Weight gain (>10 pounds) since breast cancer diagnosis independently associated with hot flash occurrence (OR 2.1; 95% CI: 1.1, 4.4) and hot flash severity (OR 2.6; 95% CI: 1.3, 5.0). Younger age and depressive symptoms were risk factors for hot flash severity.
Tane 2017 [19]	Japan	Prospective cohort	Postmenopausal. ER+, Japanese	391	Anastrozole: 100%	Multivariate analysis ^c^: BMI: OR 1.09 per unit of increase (95% CI 1.02, 1.16; *p* = 0.009). Menopausal disorders during menopause: OR 2.11 (95% CI: 1.35, 3.30; *p* = 0.001).
Umamaheswaran 2020 [20,21]	India	Prospective	Stage 0–III, postmenopausal. HR+	198	Letrozole: 100%	*CYP19A1* Haplotypes H10-TCC GTT GGGCG (0.5% vs. 3.7%, *p* = 0.042) lacking variant allele, (rs700519-T) showed protection to VMS toxicity risk. H6-GCC AGC TGGCG (7.3% vs. 2.8%, *p* = 0.037) haplotype exhibited higher VMS frequency. H3-GTC GTT GCACG (4.1% vs. 10.3%, *p* = 0.043) significantly associated with decreased VMS risk. Greater proportion of individuals with VMSs (20.5%) carried the 3-marker haplotype GCC compared with individuals without VMSs (7.9%; *p* = 0.002). Higher proportion of individuals with GTC haplotype without VMSs (22.9%) than with VMSs (10.2%).
**Interventional (n = 4)**						
Ganz 2016 [43] NSABP B-35 NCT00053898	US	RCT (anastrozole vs. tamoxifen)	Early stage, postmenopausal DCIS or mixed ductal carcinoma in situ and lobular carcinoma in situ, ER+ or PR+, with no invasive component	1193	Anastrozole: 49.6% Tamoxifen: 50.4%	VMSs (1.33 vs. 1.17; *p* = 0.011) more severe in individuals treated with tamoxifen than anastrozole. When adjusted for treatment, younger age was associated with more severe VMSs (mean severity score 1.45 for age < 60 yrs vs. 0.65 for age ≥ 60 yrs; *p* = 0.0006).
Huober 2014 [40] BIG1-98 NCT00004205	Denmark, France, Switzerland	RCT (letrozole vs. tamoxifen)	Early stage, postmenopausal HR+	4682	Letrozole: 50% Tamoxifen: 50%	The occurrence of arthralgia, myalgia, carpal tunnel, and/or VMSs was associated with treatment group, age quartile, and prior HRT use in multivariate logistic regression analysis.
Goss 2013 [45] MA.27 NCT00066573	Canada	RCT (exemestane vs. anastrozole)	Early stage, postmenopausal HR+, primary invasive	7576	Exemestane: 50% Anastrozole: 50%	White individuals reported significantly more hot flashes than non-White individuals (*p* ≤ 0.003).
Fontein 2014 [38] TEAM ^d^	The Netherlands	RCT (analyses conducted only on participants randomized to receive 5 yrs of exemestane)	Early stage, postmenopausal ER- and/or PR+	737	Exemestane: 100%	Homozygous genotypes of 2 *CYP19A1* SNPs associated with VMSs. AA genotype of rs934635: OR 2.775 (95% CI: 1.02, 7.56). TT genotype of rs7176005: OR 4.9 (95% CI: 1.02, 23.5).

AET, adjuvant endocrine therapy; BC, breast cancer; BMI, body mass index; CT, chemotherapy; DCIS, ductal carcinoma in situ; ER, estrogen receptor; NR, not reported; OR, odds ratio; PR, progesterone receptor; SNP, single-nucleotide polymorphism; VMS, vasomotor symptom; WHI, Women’s Health Initiative. ^a^ The 3-point cutoff was chosen because it represents a clinically meaningful symptom burden change. ^b^ After adjusting for age, smoking, race, current height and weight, and depressive and anxiety symptoms. ^c^ Including age, BMI, years from menopause, and menopausal disorders during menopause. ^d^ Patients were selected from the cohort of Dutch TEAM patients.

## Data Availability

The data used in this SLR were extracted from the existing studies cited in the manuscript and are available in the public domain; however, some are behind a paywall and require a fee for access. The data extracted from each study are described in the tables.

## Supplementary Materials

The following supporting information can be downloaded at: https://www.mdpi.com/article/10.3390/jcm14082601/s1. References [53,54] are cited in Supplementary Materials.
