# Supplementary material for: The Burden of Illness of Treatment-Induced Vasomotor Symptoms in Individuals with Breast Cancer: A Systematic Literature Review
Source: J Clin Med. 2025 Apr 10;14(8):2601. doi: 10.3390/jcm14082601 (PMC12027725; doi:10.3390/jcm14082601)
Supplement: Supplementary file 1 [file jcm-14-02601-s001.zip › jcm-3410281-supplementary.pdf]

Table S1. Search strategy with results for Embase and MEDLINE via Ovid.com.

Search	Query
1	adrenergic system.ti,ab.
2	exp adrenergic system/
3	Sympathetic Nervous System.mp.
4	vasomotor.ti,ab.
5	or/1-4
6	exp breast tumor/ use oemezd
7	Breast Neoplasms/ use medall
8	breast cancer.ti,ab.
9	or/6-8
10	5 and 9
11	limit 10 to yr="2010-2023"
12	conference abstract.pt.
13	limit 12 to yr="1974-2019"
14	11 not 13
15	remove duplicates from 14

Table S2. Study characteristics of included studies.

Study	Country	Study Design	Data Source	Time Period	Study Objective(s)	Sample Size	Menopausal Status	Stage of BC	Tamoxifen/AI Use
**Observational studies (n = 22)**									
Chaudhry 2019 [29]	US	Retrospective cohort	Chart review	NR	To evaluate the tolerability, treatment side effects, and the discontinuation rate of AIs in women over the age of 75 with early-stage BC	55	Post	Early	Adjuvant AIs
Chen 2013 [11]	China	Prospective cohort	Primary data collection	2002–2006	To examine the association of menopausal symptoms experienced by BC patients with disease-specific outcomes such as recurrence in an Asian population	4842 (56% with menopause symptoms)	Pre/post	0–III	Tamoxifen (52%)
Cole 2022 [28]	Canada	Decision model	Cross-sectional survey data	NR	To create a gradient-boosted decision tree model to identify factors that predict patients at risk of severe VMSs	360	Pre/post	Early	Endocrine therapy: 88.6%
De Valois 2010 [33]	England	Prospective cohort	National Health Service cancer treatment center	2003–2004	To evaluate the feasibility of the National Acupuncture Detoxification Association 5-point ear acupuncture protocol to reduce HFNS and improve physical	50	NR	Early	Tamoxifen

					and emotional well-being for women receiving adjuvant treatments for BC				
De Valois 2012 [34]	England	Prospective cohort	National Health Service cancer treatment center in Southern England	2001–2002	To explore whether traditional acupuncture could reduce HFNS frequency, improve physical and emotional well-being, and improve perceptions of HFNS	52	NR	Early	Tamoxifen
Egawa 2020 [12]	Japan	Prospective cohort	Primary data collection	NR	To examine the influence of AEs on prognosis in Japanese postmenopausal patients with BC treated with adjuvant anastrozole	391	Post	NR	Adjuvant anastrozole
Ernst 2021 [30]	US	Retrospective cohort	Cancer registry	2012–2017	To assess the current practice patterns regarding AI-associated symptoms in BC patients	179 (of which 89 had VMSs)	Pre/post	I–III	For the whole study population: Anastrozole: 85.5% Exemestane: 4.47% Letrozole: 10.1%
Hu 2022 [31]	US	Retrospective cohort	Tennessee cancer registry; Medicare and Medicaid claims	2007–2015	To investigate the association between symptom burden and AET adherence differences by race	559	NR	Early	AET drug types during 1st year: AI (anastrozole, exemestane, and letrozole): 57.7% Tamoxifen: 21.4% AI and tamoxifen: 20.8%
Lintermans 2016 [13]	Belgium	Prospective cohort	Primary data collection	NR	To evaluate whether genetic variants may predict endocrine therapy-related musculoskeletal pain and hot flashes	254	Post	Early	Tamoxifen: 37.4% AI (letrozole, anastrozole, or exemestane): 62.6%
Jeong 2013 [14]	Korea	Prospective cohort	East–West Medical Center at Daegu Catholic University Medical Center	2011–2012	To assess the feasibility and safety of acupuncture for treatment of hot flashes in Korean patients with BC receiving anti-estrogen therapy	10	Pre/post	NR	Tamoxifen: 90% Anastrozole: 10%
Li 2020 [15]	US	Prospective cohort	R01-CA107408, UPMC Hillman Cancer Center	NR	To identify symptom clusters that are present at four time points from preadjuvant therapy up to 18 mos of adjuvant therapy among postmenopausal women with BC	354	Post	Early	Anastrozole

Marino 2016 [22]	Australia	Cross-sectional	Primary data collection	2003–2010	To determine the association between adjuvant chemotherapy for BC and menopausal symptoms, sexual function, and quality of life	804	Pre/post	Any	Of those using anti-estrogen endocrine therapy: Tamoxifen: 59.8% Anastrozole: 23.3% Letrozole: 10.5% Goserelin and exemestane: 1.5%
Morgan 2017 [23]	UK	Cross-sectional	National Cancer Research Institute UK Breast Clinical Studies Group Working Party on Symptom Management subgroup	NR	To gauge current clinical practice for the management of hot flushes and night sweats by surveying BC patients, GPs, and healthcare professionals	NR	NR	NA	NR
Nugent 2016 [16]	US	Prospective cohort	University of Pittsburgh Cancer Institute Magee Women's BC Program	2005–2012	To examine differences in mood changes for BC survivors during the 1st year of AI therapy between occupational classifications (Skill Level 4 vs. Skill Levels 1–3) before therapy; and to examine differences in changes in reported symptom burden during the 1st year of AI therapy between those employed at Skill Level 4 and those employed at lower Skill Levels (Skill Level 1–3) before therapy	49	Post	I–IIIa	Anastrozole
Panjari 2011 [17]	Australia	Prospective cohort	Primary data collection	2004–2006	To evaluate the impact of the first diagnosis of invasive BC and its treatment, menopausal symptoms, and body image on sexual function	1588	Pre/post	Any	Tamoxifen: 43.6% Anastrozole: 25.9% Letrozole: 2.2%
Phligbua 2018 [24]	Thailand	Cross-sectional	Primary data collection	May-Sept 2016	To evaluate the psychometric properties of the Thai MENQOL instrument in menopausal Thai women with a history of BC	290	Post	Any	Tamoxifen: 77.6% AIs: 22.4%
Reeves 2018 [18]	US	Prospective cohort	Life and Longevity After Cancer Study, an ancillary study of the	2013	To evaluate predictors of VMSs	3595	Post	Localized, regional, or distant	Adjuvant hormone therapy: 66.9%

			Women's Health Initiative						
Sánchez-Borrego 2013 [32]	Spain	Position statement	NR	NR	NR	NR	Post	NA	NR
Sayakhot 2011 [25]	Australia	Cross-sectional	Primary data collection	2008–2010	To investigate menopausal symptoms, psychological symptoms, sexual function, and body image experienced by younger Australian women with BC and to examine the effect of different BC treatments on menopausal, physical, and psychological symptoms	114	Peri/post	Nonmetastatic	AI/tamoxifen: 76%
Su 2010 [26]	US	Cross-sectional	Survey (single center)	April-Oct 2007	To describe the characteristics of self-reported hot flashes in postmenopausal women with early-stage BC receiving AIs and to identify the demographic and clinical factors that are associated with hot flashes	300	Post	I–III	Anastrozole: 58%<br>Letrozole: 23%<br>Exemestane: 19%
Tane 2017 [19]	Japan	Prospective cohort	Study of Arthralgia and VMSs in Japanese Post-menopausal Women Treated with Anastrozole: SAVS-JP, UMIN000002455	2009–2012	To identify risk factors for AI-related VMSs in Japanese women	391	Post	NR	Adjuvant anastrozole
Umamaheswaran 2020 [20,21]	India	Observational, prospective	Outpatient Department, General Surgery and Medical Oncology, Jawaharlal Institute, Pondicherry	2009–2015	To determine whether SNPs in the *CYP19A1* and in the *TCL1A* genes are associated with adjuvant letrozole-induced 'specific' musculoskeletal AEs and VMSs in postmenopausal HR+ BC patients.	198 (of which 57 had VMSs)	Post	NR	Letrozole
Yeo 2020 [27]	China (Hong Kong)	Cross-sectional	Primary data collection	2008–2011	To assess chemotherapy-associated symptoms that were related to long-term menstrual disturbances and to evaluate their	280	Pre	I–III	Adjuvant tamoxifen: 41.1%

					effect on BC-specific QoL				
**Interventional studies (n = 15)**									
Fallowfield 2012 [35] ISRCTN11883920	UK	RCT	Intergroup; Exemestane Study	NR	To report on- and post-treatment QoL impact in the Intergroup Exemestane Study that demonstrated improved survival for postmenopausal women with ER + /unknown primary BC	582	Post	NR	Patients switched to exemestane after 2–3 yr tamoxifen and compared with those continuing tamoxifen to complete 5 yr therapy
Fogel 2020 [42] NCT02513329	US	Cross-sectional (BL data from a trial)	NCT02513329	NR	To further the understanding of menopause-related risk factors for cognitive difficulties in BC patients participating in an RCT of SGB and to identify modifiable risk factors that can be targeted in future studies	30	Pre/post (77% post)	NR	AIs: 63.33%<br>Tamoxifen: 36.7%
Forbes 2016 [47] ISRCTN37546358	New Zealand, Australia, UK, Italy, Germany, France, Belgium, Austria	RCT	Primary data collection	2003–2012	To compare the efficacy of anastrozole with that of tamoxifen in postmenopausal women with HR+ DCIS	2938	Post	DCIS	Anastrozole: 49.3%<br>Tamoxifen: 50.7%
Ganz 2016 [43] NSABP B-35 NCT00053898	US	RCT	Primary data collection	2003–2006	To assess the effect of anastrozole versus tamoxifen on quality of life and symptoms	1193	Post	Early	Anastrozole: 592<br>Tamoxifen: 601
Haest 2012 [36]	Belgium	Uncontrolled prospective study	Primary data collection	2008–2009	To assess the acceptability and safety of SGB treatment as well as its efficacy after 1 month of follow-up	9 (pilot study)<br>25 (main study)	Post	Early	Tamoxifen: 17<br>Oral AI: 4
Haute Autorité de Santé 2015 [53]	France	Appraisal-report	Trial data	NR	Renewal of drug registration	NR	Post	Early/advanced	NR
Huober 2014 [40] BIG1-98 NCT00004205	Denmark, France, Switzerland	RCT	BIG 1–98 NCT00004205	NR	To assess the relationship between the incidence of VMSs and arthralgia/myalgia symptoms and treatment outcomes for women with postmenopausal HR+ BC who received	4682	Post	Early	Letrozole: 2338<br>Tamoxifen: 2344

					adjuvant letrozole or tamoxifen				
Liljegren 2012 [37]	Sweden	RCT	Primary data collection	2002–2005	To evaluate true acupuncture to control acupuncture in BC patients treated with adjuvant tamoxifen suffering from hot flushes and sweating	74	Pre/post	NR	Tamoxifen
Goss 2013 [45]<br>Stearns 2015 [46]<br>MA.27<br>NCT00066573	Canada	RCT	NCIC Clinical Trials Group MA.27 (NCT00066573)	2003–2010	To compare efficacy and safety of exemestane to anastrozole for 1st-line adjuvant treatment of hormone-dependent early BC in postmenopausal women	7576	Post	Early	Exemestane: 3789<br>Anastrozole: 3787
Saha 2017 [48]<br>NCT00066690<br>NCT00066703	International	RCT	SOFT (NCT00066690); TEXT (NCT00066703)	2003–2011	To describe benefits and toxicities of adjuvant endocrine therapies in women younger than 35 yrs with BC	582	Post	Early	SOFT: exemestane + OFS vs. tamoxifen + OFS vs. tamoxifen alone<br>TEXT: exemestane + OFS vs. tamoxifen + OFS
Fontein 2012 [41]<br>TEAM<br>BOOG2006-04	Belgium, the Netherlands	RCT (Subgroup analysis)	Dutch and Belgian patients treated with exemestane, in the TEAM trial	NR	To assess the relationship between the occurrence and types of AEs and efficacy, as well as the association between specific AEs (hot flashes and musculoskeletal AEs) and quantitative ER and PR expression based on the Allred score	1485	Post	Early	Exemestane
Fontein 2013 [49]<br>TEAM<br>BOOG2006-04	The Netherlands, Germany, Belgium, France, Greece, Japan, UK, Ireland, US	RCT	TEAM trial	NR	To investigate the relationship between survival outcomes and specific AEs including VMSs, musculoskeletal AEs, and vulvovaginal symptoms in postmenopausal patients with ER- and/or PR+ BC	9325	Post	Early	Exemestane: 2389<br>Tamoxifen to exemestane: 2304
Fontein 2014 [38]<br>TEAM<br>BOOG2006-04	The Netherlands	RCT (Subgroup analysis)	Dutch patients randomized exemestane in the TEAM trial	NR	To investigate the relationship between musculoskeletal AEs and VMSs and SNPs in the *CYP19A1* gene in postmenopausal, HR+ early BC patients	737	Post	Early	Exemestane

					treated with adjuvant exemestane for 5 yrs				
Fontein 2017 [39] TEAMIIA BOOG2006-04	The Netherlands	Single-arm trial	Primary data collection; TEAMIIA trial	2007–2012	To determine if specific AEs may be associated with tumor response to AIs in the neoadjuvant setting in women with ER+ invasive breast adenocarcinoma	102	Post	I–III	Exemestane
Vrselja 2022 [50] NCT03518138	Australia, New Zealand, US	RCT	Primary data collection	2018–2020	To assess the efficacy and safety of Q-122 (a non-hormonal compound) in women with BC taking oral AET and experiencing VMSs	131	Pre/post	NR	Q-122 group: Tamoxifen: 57% AI: 42% Toremifene: 2% Placebo group: Tamoxifen: 59% AI: 41%
Walker 2010 [44]	US	RCT	Primary data collection	2004–2007	To evaluate the effect of acupuncture in reducing/eliminating VMSs in patients with BC who received anti-estrogen hormone therapy as compared with venlafaxine and to determine whether acupuncture has fewer adverse effects	50	Pre/post	0–III	Tamoxifen or anastrozole

AE, adverse event; AET, adjuvant endocrine therapy; AI, aromatase inhibitor; BC, breast cancer; DCIS, ductal carcinoma in situ; GP, general physician; HFNS, hot flashes and night sweats; HR+, hormone receptor-positive; MENQOL, Menopause-Specific Quality of Life Questionnaire; NCIC, National Crime Information Center; NR, not reported; OFS, ovarian function suppression; PR, progesterone receptor; QOL, quality of life; RCT, randomized controlled trial; ; SGB, stellate ganglion block; SNP, single-nucleotide polymorphism; UK, United Kingdom; US, United States; VMS treatment-induced vasomotor symptom.

Table S3. Prevalence and incidence of VMS in included studies.

Study	Country	Study design	Population	BC maintenance treatment(s)	N	Reported prevalence/incidence of VMS
**Observational (n=12)**						
Chaudhry 2019[29]	US	Retrospective cohort	Early stage, postmenopausal	Adjuvant AI: 100%	55	Incidence<br>Hot flashes: 7.3%
Chen 2013[11]	China	Prospective cohort	Early stage, pre/postmenopausal, ER+	Tamoxifen	2,093	Prevalence<br>Hot flashes: 50.8%<br>Night sweats: 38.9%
Ernst 2021[30]	US	Retrospective cohort	Stage I-III, pre/postmenopausal Estrogen and/or progesterone +	Anastrozole: 85.5% Exemestane: 4.47%	179	Prevalence (BL)<br>VMS: 50%

				Letrozole: 10.1%		
Li 2020[15]	US	Prospective cohort	Early stage, postmenopausal, newly diagnosed	Anastrozole: 100%	354	Prevalence (BL)<br>Night sweats: 41.5%<br>Hot flashes 49.7%<br>Prevalence (18 mos; n=156)<br>Night sweats: 48.0%<br>Hot flashes 64.1%
Lintermans 2016[13]	Belgium	Prospective cohort	Early stage, postmenopausal, starting AI or tamoxifen	Tamoxifen: 37.4%<br>AI (letrozole, anastrozole, or exemestane): 62.6%	254	Prevalence (BL)<br>Hot flashes<br>AI: 80%<br>Tamoxifen: 77%
Marino 2016[22]	Australia	Cross sectional	Any stage, pre/postmenopausal	Of those using anti-estrogen endocrine therapy (400 out of 803 study participants):<br>Tamoxifen: 59.8%<br>Anastrozole: 23.3%<br>Letrozole: 10.5%<br>Goserelin and exemestane: 1.5%	400	Prevalence<br>Hot flashes in the previous 24 hours:<br>Tamoxifen: 77%<br>AI (anastrozole, letrozole, or exemestane): 79%
Panjari 2011[17]	Australia	Prospective cohort	Any stage, pre/postmenopausal<br>Individuals recruited within 12 mos of their first diagnosis with invasive BC	Among the 1,588 individuals who completed follow-up questionnaire<br>No therapy: 28.2%<br>Tamoxifen: 43.6%<br>Anastrozole: 25.9%<br>Letrozole: 2.2%	Completed the follow-up questionnaire: 1,588	Prevalence at 12 mos after enrolment questionnaire<br>VMS: 77% (of 1,011 individuals in the analysis)
Reeves 2018[18]	US	Prospective cohort	Localized, regional, or distant, postmenopausal. Invasive	Adjuvant hormone therapy: 66.9%	3,595	Prevalence among those treated with adjuvant hormone therapy (n=2,406)

						VMS: 26.6%
Sayakhot 2011[25]	Australia	Cross sectional	Nonmetastatic, peri/postmenopausal	AI: 33%<br>Tamoxifen: 43%<br>No adjuvant endocrine therapy: 24%	114	Individuals on AI (n=81) reported significantly higher VMS compared with individuals not receiving AI[a] (n=24)<br>Greene Climacteric Scale scores (n=105): 3.50 vs 2.56; P=0.01<br>Prevalence (n= 105)<br>Hot flashes: 83%<br>Night sweats: 77%
Su 2010[26]	US	Cross sectional	Stage I-II, postmenopausal HR+	Anastrozole: 58%<br>Letrozole: 23%<br>Exemestane: 19%	300	Prevalence<br>Hot flashes: 59%
Tane 2017[19]	Japan	Prospective cohort	Postmenopausal ER+	Adjuvant anastrozole 100%	391	Prevalence prior to anastrozole treatment (BL)<br>Hot flashes: 20.5%<br>Night sweats: 15.1%<br>Cold sweats: 8.2%<br>Prevalence (12 mos, n=305)<br>Hot flashes: 32.5%<br>Night sweats: 22.6%<br>Cold sweats: 22.0%<br>Incidence over the course of 12 mos<br>Hot flashes: 34.8%<br>Night sweats: 29.3%<br>Cold sweats: 28.7%
Umamaheswaran 2020[20]	India	Prospective cohort	Stage 0-III, postmenopausal HR+	Letrozole 100%	198	Prevalence (BL)<br>VMS: 28.8%
**Interventional (n=6 independent studies)**						
Forbes 2016[47] ISRCTN37546358	New Zealand, Australia, UK, Italy, Germany, France, Belgium, Austria	RCT (anastrozole vs tamoxifen)	DCIS Dx, postmenopausal	Anastrozole: 49.3%<br>Tamoxifen: 50.7%	2,938	Incidence (at any time during the 5 yrs of treatment)<br>Hot flashes:<br>Anastrozole: 56%<br>Tamoxifen: 60%
Goss 2013[45] MA.27 NCT00066573	Canada	RCT (exemestane vs anastrozole)	Early stage, postmenopausal HR+, primary invasive	Exemestane: 50%<br>Anastrozole: 50%	7,576	Incidence (at any time during the 5 yrs of treatment)<br>Hot flashes:

						Exemestane: 55%<br>Anastrozole: 56%
Stearns 2015[46]<br>MA.27<br>NCT00066573	Canada	RCT (exemestane vs anastrozole)	Early stage, postmenopausal HR+, primary invasive	Exemestane: 50%<br>Anastrozole: 50%	5,645[b]	Incidence<br>VMS incidence at 6 mos of treatment: 17.8%<br>VMS incidence at 12 mos of treatment: 34.8%
Fontein 2013[49]<br>TEAM	The Netherlands, Germany, Belgium, France, Greece, Japan, UK, Ireland, US	RCT (exemestane for 5 yrs or tamoxifen for 2.5 to 3 yrs, followed by exemestane for 2.5 to 2 yrs)	Early stage, postmenopausal ER- and/or PR+	Exemestane: 50.3%<br>Tamoxifen to exemestane: 49.7%	9,325	Incidence<br>VMS within the 1st yr of treatment: 32.2%
Fontein 2014[38]<br>TEAM[d]	The Netherlands	RCT (analyses conducted only on participants who received 5 yrs of exemestane and who had available tumor tissue)	Early stage, postmenopausal ER- and/or PR+	Exemestane 100%	737	Prevalence (BL)<br>VMS: 22.1%
Fontein 2017[39]<br>TEAMIIA<br>BOOG2006-04	The Netherlands	Single-arm study	Stage I-III, postmenopausal ER+, invasive adenocarcinoma	Exemestane 100%	102	Incidence (at any time during the 6 mos of study treatment)<br>VMS: 25.4%
Vrselja 2022[50]<br>NCT03518138	Australia, New Zealand, US	RCT (Q-122 vs placebo)	Pre/postmenopausal and experiencing at least 50 self-reported moderate to severe VMS per week	Q-122[d] group:<br>Tamoxifen: 57%<br>AI:42%<br>Toremifene: 2%<br>Placebo:<br>Tamoxifen: 59%<br>AI:41%	131	Incidence (commencing on or after random assignment to study medication, treatment lasted 28 days)<br>Hot flashes treatment-emergent AEs<br>Q-122 group: 8%<br>Placebo group: 2%

AI, aromatase inhibitor; BC, breast cancer; BL, baseline; DCIS, ductal carcinoma in situ; Dx, diagnosis; ER, estrogen receptor; HR, hormone receptor; Mo(s), month(s); PR, progesterone receptor; RCT, randomized controlled trial; VMS, vasomotor symptoms. [a]Measured with questions 18 to 20 of the Greene Climacteric Scale [54]. [b]Subset of individuals without VMS or Grade 3/4 musculoskeletal symptoms at baseline. [c]Patients were selected from the cohort of Dutch TEAM patients. [d]Q-122 is an experimental nonhormonal small molecule inhibitor of KNDy neuron activation.

Table S4. Frequency and severity of VMS.

Study	Country	Study design	Population	BC maintenance treatment(s)	N	Reported VMS frequency and/or severity
**Observational (n=9)**						
Cole 2022[28]	Canada	Decision model	Early stage, pre/postmenopausal	Endocrine therapy (89%)	295	Frequency<br>Hot flashes per wk at BL: median (IQR) 15 (5–35)
De Valois 2012[34]	England	Prospective cohort (treatment evaluated: National Acupuncture Detoxification Association 5-point ear acupuncture protocol to reduce hot flashes and night sweats)	Early stage, menopausal status NR	Tamoxifen: 100%	47	Frequency (hot flashes and night sweats per day over the 14 days, mean [SD])<br>BL: 10.7 (4.8)<br>End of treatment: 7.7 (4.7)
Hu 2022[31]	US	Retrospective cohort	Early stage, menopausal status NR<br>HR+ BC	AET drug types during 1st yr:<br>AI (anastrozole, exemestane, and letrozole): 57.7%<br>Tamoxifen: 21.4%<br>AI and tamoxifen: 20.8%	559	Severity<br>Mean BL severity and mean changes during the first yr of AET for each individual symptom[a]<br>• Sweating<br>BL: 1.40<br>12 mos: 2.73 [p<0.05]<br>• Hot Flashes/flashes<br>BL: 1.60<br>12 mos: 3.04 [p<0.05]<br>• Trouble sleeping<br>BL: 2.2<br>12 mos: 3.4 [p<0.05]<br>• Daytime sleepiness<br>BL: 1.33<br>12 mos: 2.27 [p<0.05]<br>• Fever or chills<br>BL: 0.61<br>12 mos: 1.22 [p<0.05]

Jeong 2013[14]	Korea	Prospective cohort (treatment evaluated 12 sessions of acupuncture, delivered 3 times a week for 4 weeks)	Stage NR, Pre/postmenopausal	Tamoxifen: 90%<br>Anastrozole: 10%	10	24 hr hot flashes frequency, mean (SD):<br>BL: 9.3 (9.7)<br>End of treatment: 1.5 (1.7)<br><br>VAS, mean, SD<br>BL: 71.5 (11.3)<br>End of treatment: 19.5 (13.2)<br><br>24 hr hot flash severity, mean (SD):<br>BL: 2.8 (0.8)<br>End of treatment: 1.1 (0.7)
Li 2020[15]	US	Prospective cohort	Early stage, Postmenopausal Newly diagnosed	Anastrozole: 100%	BL: 354<br>6 mos: 288<br>12 mos: 205<br>18 mos: 156	Occurrence Rate % (n) individuals:<br>*Night sweats, %*<br>BL: 41.5<br>6 mos: 50.0<br>12 mos: 50.2<br>18 mos: 48.0<br>*Hot flashes, %*<br>BL: 49.7<br>6 mos: 63.1<br>12 mos: 70.5<br>18 mos: 64.1<br><br>Severity (via BCPT Symptom Checklist)<br>*Night sweats, mean (SD)*<br>BL: 17.73 (26.04)<br>6 mos: 23.44 (29.98)<br>12 mos: 23.17 (28.97)<br>18 mos: 20.19 (25.89)<br>*Hot flashes, mean (SD)*<br>BL: 22.18 (28.08)<br>6 mos: 28.91 (28.84)<br>12 mos: 32.60 (29.21)<br>18 mos: 29.49 (28.47)
Morgan 2017[23]	UK	Cross sectional	Individuals with BC	NR[b]	NR[b]	Severity

						31% of individuals said hot flashes and night sweats were severe enough for them to consider stopping endocrine therapy
Phligbua 2018[24]	Thailand	Cross sectional	Any stage, postmenopausal; experiencing hot flashes	Tamoxifen: 77.6%<br>AIs: 22.4%	290	<u>Severity</u><br>*MENQOL (ranging from 1, no symptoms to 8, extremely bothersome)*<br>*Mean (SD)*<br>Vasomotor subscale, including: 5.95 (3.86)<br>Hot flashes: 2.26 (1.36)<br>Night sweats: 1.46 (1.46)<br>Sweating: 2.23 (1.90)
Su 2010[26]	US	Cross sectional	Stage I-II, postmenopausal HR+	Anastrozole: 58%<br>Letrozole: 23%<br>Exemestane: 19%	300	<u>Hot flashes severity</u><br>Mild: 27%<br>Moderate, severe, very severe: 32%<br><u>Perceived change in hot flashes since AI therapy</u><br>Significant worsening: 25%<br>Little or no change: 69%<br>Significant improvement: 6%
Tane 2017[19]	Japan	Prospective cohort	Postmenopausal ER+, Japanese individuals	Anastrozole:100%	391	<u>Severity (% of individuals)</u><br>Maximal grades of VMS developed during anastrozole treatment in individuals without VMS at BL; Grade 1: mild; Grade 2: moderate; Grade 3: severe<br>*Hot flashes*<br>Grade 1: 84.2%<br>Grade 2: 15.8%<br>Grade 3: 0%<br>*Night Sweats*<br>Grade 1: 86.0%

						Grade 2: 10.0%<br>Grade 3: 4.0%<br>*Cold Sweats*<br>Grade 1: 79.2%<br>Grade 2: 19.8%<br>Grade 3: 1.0%
**Interventional (n=6)**						
Fogel 2020[42]<br>NCT02513329	US	RCT (participants were participating in an RCT of stellate ganglion blockade. This study analysed data from the baseline visit of this larger, parent trial)	Stage NR, pre/postmenopausal with moderate-to-severe VMS (≥28 per wk reported on a diary)	AIs: 63.33%<br>Tamoxifen:36.7%	30	*VMS (daily count) at BL, mean (SD)*<br>Physiologic (via ambulatory skin conductance monitors):<br>Total: 20.30 (10.44)<br>Awake: 15.23 (7.98)<br>Sleep: 5.08 (4.61)<br>*Subjective (via diary/button presses)*<br>Total: 6.47 (5.40)<br>Awake: 5.03 (4.29)<br>Sleep: 1.43 (2.27)
Ganz 2016[43]<br>NSABP B-35<br>NCT00053898	US	RCT (anastrozole vs tamoxifen)	Early stage, postmenopausal DCIS or mixed ductal carcinoma in situ and lobular carcinoma in situ, ER+ or PR+, with no invasive component	Anastrozole: 49.6%<br>Tamoxifen: 50.4%	1,193	Severity of VMS (mean score, measured by the BCPT symptom scale)<br>6 mos<br>• Tamoxifen 1.18 vs Anastrozole 1.51; P<0.0001<br>12 mos<br>• Tamoxifen 1.65 vs Anastrozole 1.41; P=0.0007<br>30 mos<br>• Tamoxifen 1.30 vs Anastrozole 1.10; P=0.0085<br>36 mos<br>• Tamoxifen 1.24 vs Anastrozole 1.09; P=0.0444<br>Overall Severity<br>• Tamoxifen 1.33 vs Anastrozole 1.17; P=0.011
Liljegren 2012[37]	Sweden	RCT[c] (acupuncture vs non-insertive stimulation at non-	Stage NR, pre/postmenopausal	Tamoxifen:100%	74	Frequency<br>BL, mean (SD) per 24 hours<br>*Hot flashes (at BL)*<br>• Acupuncture: 8.4 (5.5)

		acupuncture points [control])				• Control: 7.1 (4.4)<br>*Hot flashes (at 18 wks)*<br>• Acupuncture: 5.6 (4.7)<br>• Control: 5.4 (4.5)<br>*Sweating (at BL)*<br>• Acupuncture: 8.4 (4.9)<br>• Control: 6.3 (4.3)<br>*Sweating (at 18 wks)*<br>• Acupuncture: 5.4 (4.3)<br>• Control: 5.2 (4.7)
Fontein 2014[38] TEAM[d]	The Netherlands	RCT (analyses conducted only on participants randomized to receive 5 yrs of exemestane)	Early stage, postmenopausal ER- and/or PR+	Exemestane:100%	737	Severity<br>Mild: 69.1%<br>Moderate/severe: 30.9%
Vrselja 2022[50] NCT03518138	Australia, New Zealand, US	RCT (Q-122 100 mg vs placebo)	Stage NR, pre/postmenopausal	Q-122 arm:<br>Tamoxifen: 57%<br>AI:42%<br>Toremifene: 2%<br>Placebo arm:<br>Tamoxifen: 59%<br>AI:41%	131	• Moderate and severe VMS-SS mean LSM % CFB (over 28-d treatment period): Q-122: -39% [95% CI -46 to -31]; placebo: -26% [-33 to -18]; P=0.018<br>• Total VMS-SS mean LSM % CFB: Q-122: -35% [95% CI -42 to -29]; placebo -25% [-31 to -18]; P=0.022
Walker 2010[44]	US	RCT (12 wks of acupuncture vs 12 wks of venlafaxine at 37.5 mg for 1 wk then 75 mg for 11 wks)	Stage 0-III pre/postmenopausal with 14 hot flashes per week	Tamoxifen or arimidex (100%)	50	Frequency<br>Hot flash frequency (mean as a % of BL)<br>• Acupuncture group:<br>Pretreatment: 100<br>Post-treatment: 50<br>3 mos: 78.9<br>6 mos: 69.2<br>9 mos: 61.3<br>12 mos: 63.4<br>• Venlafaxine group:

Pretreatment: 100<br>Post-treatment: 50<br>3 mos: 69.7<br>6 mos: 83.9<br>9 mos: 65.0<br>12 mos: 74.7<br><u>Severity</u><br>Hot flash severity<br>• Acupuncture group:<br>Pretreatment: 1.6<br>Post-treatment: 1.2<br>3 mos: 1.6<br>6 mos: 1.4<br>9 mos: 1.4<br>12 mos: 1.5<br>• Venlafaxine group:<br>Pre-treatment: 1.8<br>Post-treatment:1.4<br>3 mos: 1.5<br>6 mos: 1.6<br>9 mos: 1.4<br>12 mos: 1.5

AI, aromatase inhibitor; AET, adjuvant endocrine therapy; BCPT, Breast Cancer Prevention Trial; BL, baseline; CFB, change from baseline; ER, estrogen receptor; LSM, least square means; MENQOL, Menopause-Specific Quality of Life Questionnaire; NR, not reported; PR, progesterone receptor; RCT, randomized controlled trial; VAS, visual analog scale; VMS, vasomotor symptoms; VMS-SS, weekly vasomotor symptom severity score. [a]Individual symptom items scored on a 0 to 10 scale, where 0 indicates no problem and 10 indicates as bad as possible. BL means were reported within 2 mos before starting AET; changes from the BL were determined by the highest severity symptoms reported during the 1-yr follow-up period. [b]Data reported from the National Cancer Research Institute UK Breast Clinical Studies Group Symptom Management subgroup. [c]Individuals were randomized to acupuncture or sham acupuncture for alleviation of VMS. [d]Patients were selected from the cohort of Dutch TEAM patients.
